# Long noncoding RNA HOXC-AS3 interacts with CDK2 to promote proliferation in hepatocellular carcinoma

**DOI:** 10.1186/s40364-022-00411-2

**Published:** 2022-08-28

**Authors:** Chen Su, Weijian Wang, Jie Mo, Furong Liu, Hongwei Zhang, Yachong Liu, Xiaoping Chen, Zhibin Liao, Bixiang Zhang, Peng Zhu

**Affiliations:** 1 Hepatic Surgery Center, Tongji Hospital, Tongji Medical College, Huazhong University of Science and Technology, Wuhan, Hubei 430030 People’s Republic of China; 2 Hubei Key Laboratory of Hepato-Pancreato-Biliary Diseases, Wuhan, Hubei People’s Republic of China; 3 Key Laboratory of Organ Transplantation, Ministry of Education, Wuhan, Hubei People’s Republic of China; 4 Key Laboratory of Organ Transplantation, Chinese Academy of Medical Sciences, Wuhan, Hubei People’s Republic of China

**Keywords:** HOXC-AS3, CDK2, p21, Proliferation, Hepatocellular carcinoma

## Abstract

**Background:**

Hepatocellular carcinoma (HCC) is a type of cancer that affects the liver and has a high mortality rate. Long non-coding RNAs (lncRNAs) dysregulation can contribute to cancer occurrence and progression, although the underlying molecular pathways are mostly unclear. HOXC-AS3 was found to be considerably overexpressed in HCC in this investigation. The goal of this work was to look into the involvement of HOXC-AS3 in HCC and the various molecular pathways that underpin it.

**Methods:**

Normal liver and paired HCC tissues from HCC patients were used to evaluate HOXC-AS3 expression by qRT-PCR. The role of HOXC-AS3 in HCC was assessed both in vitro and in vivo. RNA pulldown, RIP and co-IP were used to demonstrate the potential mechanism by which HOXC-AS3 regulates the progression of HCC.

**Results:**

Using qRT-PCR, it was discovered that HOXC-AS3 was substantially expressed in HCC. In vitro and in vivo, overexpression of HOXC-AS3 aided proliferation and cell cycle progression. HOXC-AS3 interacted with CDK2 to facilitate CDK2’s decreased binding to p21, resulting in enhanced CDK2 activity, which promoted the phosphorylation of Rb and the progression of HCC.

**Conclusions:**

HOXC-AS3 is highly expressed in HCC and can promote the progression of HCC by interacting with CDK2. Therefore, targeting HOXC-AS3 is very likely to provide a new strategy for the treatment of HCC and for improving patient prognosis.

**Supplementary Information:**

The online version contains supplementary material available at 10.1186/s40364-022-00411-2.

## Background

Hepatocellular carcinoma (HCC) is one of the most common malignancies and the fourth leading cause of cancer-related mortality worldwide [1]. Surgical resection, radiation, chemotherapy, liver transplantation, molecular targeted therapy, and immunotherapy are not optimal treatments for HCC [2, 3]. The lack of standard clinical features and early diagnostic markers remains a significant barrier to HCC treatment [4]. As a result, new effective biomarkers and treatment targets must be identified and developed to aid in the diagnosis and analysis of HCC prognosis.

Non-coding RNAs make up 90% of the human genome, and as thousands of non-coding RNAs have been mined and annotated by scientists from sequencing data, researchers have gradually realized that the status of non-coding RNAs in diseases cannot be ignored [5]. Long non-coding RNAs (lncRNA), as one of the non-coding RNAs (ncRNA), is a type of non-coding RNA with 200 or more nucleotides [6]. It’s a class of molecules that have important biological functions and participate in various cellular processes, and many lncRNAs have been reported as biomarkers for prognosis prediction in multiple cancer types [7]. Increasing evidence has demonstrated that lncRNAs promote cancer progression by influencing chromatin remodeling, gene transcription, post-transcriptional modulation, protein localization, and intermolecular signaling pathways [8]. In HCC, previous studies have proved that dysregulation of lncRNAs participates in cancer development [9–15]. Nevertheless, the mechanisms of lncRNAs in cancers are not fully understood. Therefore, further understanding of the role of lncRNAs in HCC progression will provide a new sight for the diagnosis and treatment of HCC.

HOXC-AS3 is a newly discovered lncRNA. In the previous study, researchers have identified the full length of HOXC-AS3, and it was significantly upregulated in gastric carcinoma (GC) [14]. Currently, there is no relevant research report on the biological role of HOXC-AS3 in HCC, related molecular mechanisms, and its importance in judging the prognosis of patients. In this research, we found that the up-regulation of HOXC-AS3 predicts the poor prognosis of HCC patients. Functionally, HOXC-AS3 promotes the proliferation of HCC by accelerating the cell cycle progression (G1/S) of cancer cells. In terms of mechanism, we found that HOXC-AS3 stabilizes CDK2 at the protein level by interacting with CDK2, allowing it to maintain its carcinogenic activity. Our results clarify the role of HOXC-AS3 in the progression of HCC and believe that HOXC-AS3 may serve as a potential biomarker for the diagnosis and prognosis of HCC.

## Materials and methods

### Tissue specimens and patients

HCC tumor tissue and para cancer tissue were taken from HCC patients who had hepatectomy at Huazhong University of Science and Technology’s (HUST) Tongji Hospital (Wuhan, China). The Ethics Committee of Tongji Hospital, HUST, examined and approved all procedures, which were carried out in accordance with the Declaration of Helsinki Principles. Each patient’s written consent was obtained before the collection of specimens.

### Immunohistochemical staining

After the mouse subcutaneous tumor formation experiment, the tumor xenografts were removed from the mice, soaked in 4% formaldehyde, fixed for 1 to 24 hours, and then embedded in paraffin and made into sections. Place the slices in a 65-degree oven and bake for 30 minutes to 1 hour, then soak in xylene for 3 minutes for dewaxing, and soak in ethanol of high to low concentration for 3 minutes for rehydration. Soak the slices in 0.01 mol/l citrate buffer at 95 degrees Celsius for 30 minutes for antigen retrieval. The endogenous peroxidase activity was soaked in hydrogen peroxide (3%) at room temperature for 10 minutes in the dark, and the sections were incubated with 5% bovine serum albumin for 1 hour at 37 °C to reduce non-specific binding. Add antibody Ki-67 for immunostaining and incubate at 4 °C for 16 hours, then incubated with horseradish peroxidase (HRP)-conjugated secondary antibody for 1 hour at room temperature. DAB was added dropwise to develop color for 1 minute. After brown staining appeared, the section was immersed in distilled water to stop the reaction. The sections were then counter-stained with 1% hematoxylin for 10 minutes and then immersed in low to high concentrations of ethanol for 3 minutes for dehydration. Use a high-resolution microscope or slice scanner for image capture.

### RNA extraction and quantitative real-time polymerase chain reaction (qRT-PCR)

The FastPure Cell/Tissue Total RNA Isolation Kit V2 (Vazyme, China,RC112–01) was used to extract total RNA from tissues and cells according to the manufacturer’s instructions for RNA extraction and quantitative real-time polymerase chain reaction (qRT-PCR). A reverse-transcription system kit (Vazyme, China, R223–01) was used to complete the reverse transcription of lincRNA and mRNA, which was then evaluated by qPCR using a Universal SYBR qPCR Master Mix kit (Vazyme, China, Q711–02) Use GAPDH as an internal control to determine the mRNA level, according to the operation handbook. For relative quantification, the 2Δ-CT approach was applied. Three independent duplicates of each qRT-PCR experiment were carried out.

### Cell culture and transfection

HCC Cells 7702 (CCTCC, No.ZHYC-0297), 97H (ATCC, No. CRL-2117), HLF (ATCC, No. CRL-2105), Hep3B (ATCC, No. HB-8064), HepG2 (ATCC, No. HB-8065), PLC/PRF/5 (ATCC, No.CRL-8024) were cultured in Dulbecco’s modified Eagle’s medium (DMEM, Invitrogen) supplemented with 10% fetal bovine serum (Sijiqing, Hangzhou, China) and incubated in 5% CO2 at 37 °C.

Ribobio Technology designed and purchased antisense oligonucleotides (Asos) to knock down the HOXC-AS3 gene, as well as negative control Asos (Guangzhou, China). Lipofectamine 3000 Reagent was used to transfect Asos according to the manufacturer’s instructions (Invitrogen, USA). A full-length HOXC-AS3 was generated on the plasmid and processed into lentivirus, which was then introduced into HLF and 97H cells for stable overexpression.

### Western blot and antibodies

It was separated for 2 hours on a 10% SDS-PAGE gel before being transferred to a PVDF membrane (0.45 μm, Roche). The membrane was blocked for 1 hour at 37 °C with 5% TBST, then the primary antibody was added and incubated for 8 hours at 4 °C. The membrane was then rinsed three times with TBST before being incubated for 1 h at 37 °C with HRP-conjugated goat anti-rabbit or goat anti-mouse immunoglobulin G secondary antibody (Jackson ImmunoResearch Laboratories). TBST washed the membrane three times more. The target western blot was detected using the ECL method (Bio-Rad, USA). Proteintech provided the CDK2(22060–1-AP) and p21(10355–1-AP) antibodies used in Western blotting in this investigation, while Cell Signaling Technology provided the Rb(#9313) and p-Rb(#8516) antibodies.

### Cell proliferation assay

HCC cell proliferation was determined using the CCK-8 Cell Counting Kit (Vazyme, China, A311–01). After transfection, HLF, Hep3B, and 97H cells were seeded into 96-well plates (1000 cells/well). Then, according to the manufacturer’s protocol, the cells Culture the cells and record the 450 nm absorbance at 24, 48, 72, 96, and 120 hours respectively [16]. All experiments were performed 3 times, and the results are shown as the average value of + SD.

### Colony formation assay

Add HLF, Hep3B, and 97H cells to each well of a 6-well plate (2000 cells/plate), and continue to culture for 14 days in DMEM containing 10% FBS. Change the medium every 7 days. After 14 days, the cells were washed twice with PBS, fixed with 4% formaldehyde for 15 minutes, and stained with crystal violet for 15 minutes. Count and analyze cell clones [17].

### EdU incorporation assay

HCC cells (20,000 cells per well) are inoculated into a 24-well plate and cultured overnight. When the cell density reaches 40%, add 100 μl of 50 M EdU solution to the cells (BeyoClickTM EdU Cell Proliferation Kit with a Fluor 594, Beyotime, China,C0078S) and cultivate for another 2 hours. PBS was used to wash the cells, 4% paraformaldehyde was used to fix them, and 0.5% TritonX-100 was used to rupture them. After 30 minutes, stain the cell nucleus with 1X Hoechst 33342 solution and 100 μl 1X Apollo solution. Take images using a fluorescent microscope (EVOS FL auto imaging system, life technologies, USA) when the staining is finished to count the number of positive cells and analyze the data.

### RNA Pulldown assay

The oe-HOXC-AS3 vector and the control vector were transfected into 293 T cells. Two groups of total RNA were extracted after 48 hours, 100 nmol RNA probes were added, and the mixture was incubated at 70 °C for 5 minutes. The RNA is then gradually cooled to room temperature, allowing HOXC-AS3 to hybridize with the probe. After that, add 50 μl streptavidin magnetic beads and incubate for 30 minutes at room temperature with stirring. Unbound RNA has washed away with 20 mM Tris, and a test tube containing streptavidin magnetic beads was filled with 100 μl RNA-protein binding buffer containing 100 μg total protein. Streptavidin Magnetic Beads were washed three times with washing buffer and then incubated with 50 μl elution buffer at 37 °C for 15 minutes with stirring after incubation for 1.5 hours with rotation at 4 °C. For Western blotting, collect the supernatant.

### RNA immunoprecipitation (RIP)

RIP assay was performed to enrich Argonaute 2 (AGO2)-, Flag-P21, or Flag-CDK2-bound RNA using Magna RIP™ RNA-Binding Protein Immunoprecipitation Kit (Millipore, Germany). The antibodies used for RIP assays included antibodies against AGO2 (Abcam, UK), Flag (Sigma), and mouse IgG (Millipore). qRT–PCR was subsequently performed to analyze the enriched RNA.

### Flow cytometry

For the cell cycle investigation, HCC cells were isolated, fixed in 75% ethanol, and stored overnight at 4 °C. Following that, DNA Prep (Beckman Coulter, Brea, CA, USA) was used to stain the cells, and flow cytometry was utilized to quantify the percentage of cells at various stages based on DNA content, according to a report [18].

### Fluorescence in situ hybridization analysis (FISH)

Fluorescence in situ hybridization (FISH) study was performed using fluorescence-conjugated HOXC-AS3 probes. The target RNA was created using known nucleotide probes and the HOXC-AS3 complementary bases pairing method. By using Zeiss fluorescent confocal microscopy of nucleic acid probe hybridization of cells and tissues under qualitative and quantitative or relatively localized target RNA, the location of the target RNA may be directly visualized.

### Animal experiments

The routine source of the experimental animals was performed as previously described [19].

Forty male BALB/c nude mice (4 weeks old) were separated into four groups (Vec, HOXC-AS3, shNC, and Aso-2) and injected with 1*10^6^ HLF or 97H cells on the right side to create a subcutaneous xenograft model. The mice were euthanized after 28 days to perform cervical dislocation. The xenografts were then taken out, fixed, weighed, photographed, and stored. V (mm3) = length width^2^/2 was used to calculate the volume of the tumor. Total protein was collected from the tissues, and CDK2 expression was determined using Western blotting.

To create an orthotopic xenograft model in vivo, 28 male BALB/c nude mice (5 weeks old) were separated into four groups (Vec, HOXC-AS3, shNC, and Aso-2) and 1*10^6^ cells were injected into their livers. The livers were then fixed, photographed, conserved, and H&E stained in preparation for analysis.

### Co-Immunoprecipitation

In HEK293T cells or HCC cells, expressed labeled cell protein extracts were incubated with antibodies (Flag, HA, CDK2, or IgG as control, Sigma), and binding protein A/G beads (Pierce) for 12 h at 4 °C. The materials were analyzed by western blotting after being washed three times with an IP buffer.

### Statistical analysis

The mean ± standard error of the mean is used to express the findings (SEM). The data were analyzed using GraphPad Prism 8.3 and a two-tailed student’s t-test. P 0.05 was considered statistically significant.

## Results

### HOXC-AS3 is high expression in HCC

**Fig. 1:**
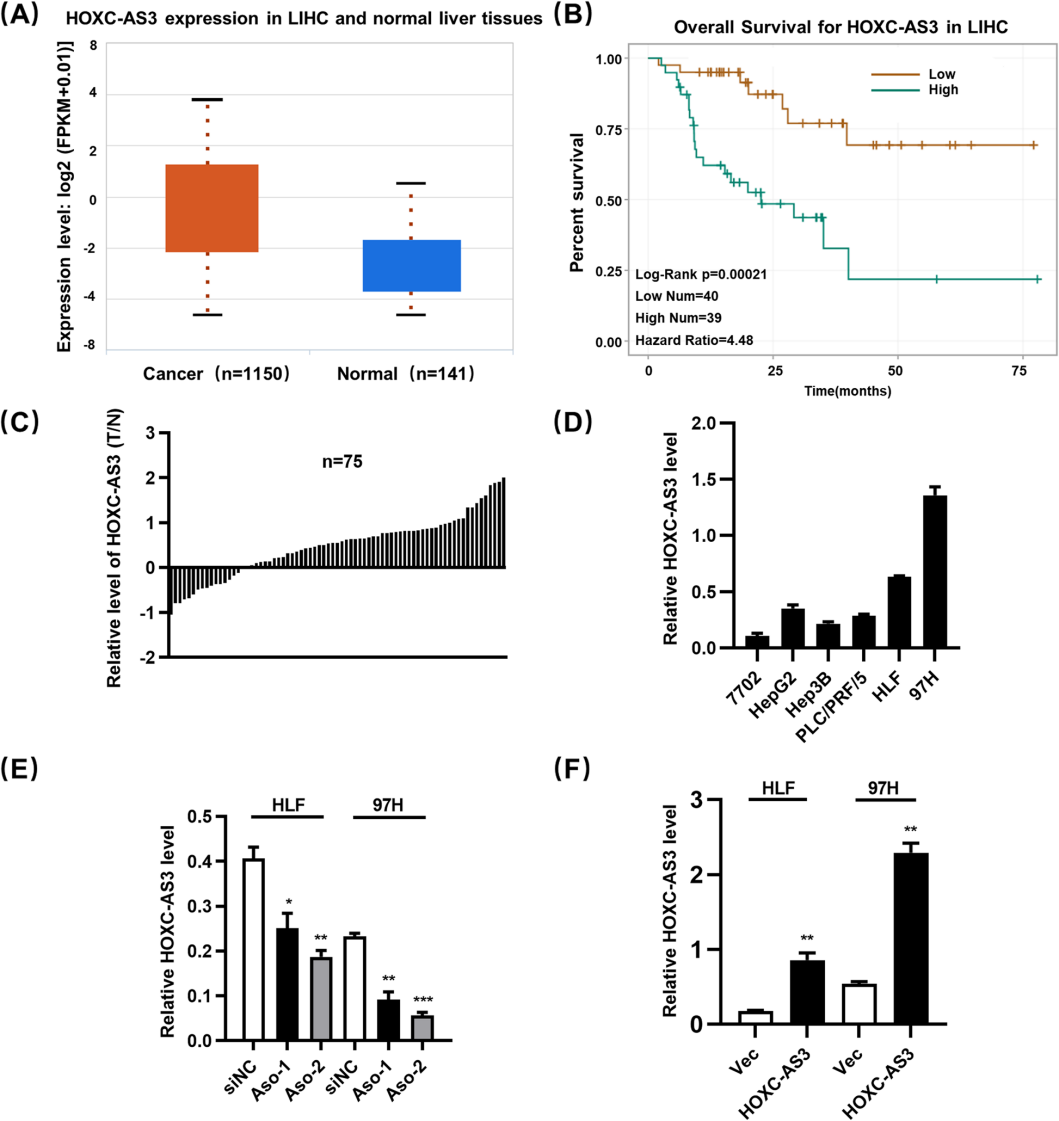
HOXC-AS3 is highly expression in HCC. **A** HOXC-AS3 expression were measured in HCC tissues and their adjacent normal tissues in StarBase (v3.0) (wilcoxon test). **B** Survival was analyzed and compared between patients with low and high levels of HOXC-AS3 in StarBase(v3.0) (log-rank test, two-sided). HR, Hazard Ratio. **C** The RNA expression level of HOXC-AS3 from 75 paired HCC samples and adjacent non-tumor liver tissues from Tongji Hospital by qRT-PCR. **D** Relative expression of HOXC-AS3 in five HCC cell lines and normal human hepatocyte 7702 by qRT-PCR. **E** Relative expression of HOXC-AS3 in HLF and 97H cells transfected with Aso-HOXC-AS3 and negative control. **F** Relative expression of HOXC-AS3 in HLF and Hep3B cells transfected with pcDNA-vec and pcDNA-HOXC-AS3 plasmids. Data represent the MEAN ± S.E.M. of three independent experiments. *P < 0.05; **P < 0.01; ***P < 0.001; ****P < 0.0001 (Student’s t test)

HOXC-AS3 is a recently discovered lncRNA with scant information on its clinical significance in cancer. In the TCGA databases-The StarBase(v3.0), we found that the expression of HOXC-AS3 was significantly higher in the HCC tissues than in the corresponding adjacent nontumor tissues (Fig. 1A). Moreover, Kaplan–Meier survival analysis showed that a high level of HOXC-AS3 was associated with the poor overall survival in HCC patients (Fig. 1B). To rule out the possibility that the uneven sample size contributed to the statistical significance, we examined HOXC-AS3 levels in 75 pairs of HCC and non-cancerous tissues. HOXC-AS3 was shown to be highly overexpressed in HCC, according to the findings (Fig. 1C). Following that, we looked for HOXC-AS3 expression in HCC cell lines (HepG2, Hep3B, ALEX, HLF, and 97H), and the qRT-PCR results showed that HOXC-AS3 was strongly expressed in HCC cells compared to the human hepatocyte cell line 7702. (Fig. 1D). These findings suggested that HOXC-AS3 may play a role in HCC carcinogenesis. To confirm its role in HCC progression, we knocked down HOXC-AS3 expression in HCC cell lines (HLF and 97H) using antisense oligonucleotides (Aso). The knockdown efficiency was verified using qRT-PCR (Fig. 1E). And we also constructed HCC cell lines (Hep3B and HLF) stably overexpressing HOXC-AS3 and control cell lines by transfecting with lentivirus. The overexpressed efficiency was verified using qRT-PCR (Fig. 1F).

### HOXC-AS3 promotes HCC cell proliferation and cell cycle progression in vitro

**Fig. 2:**
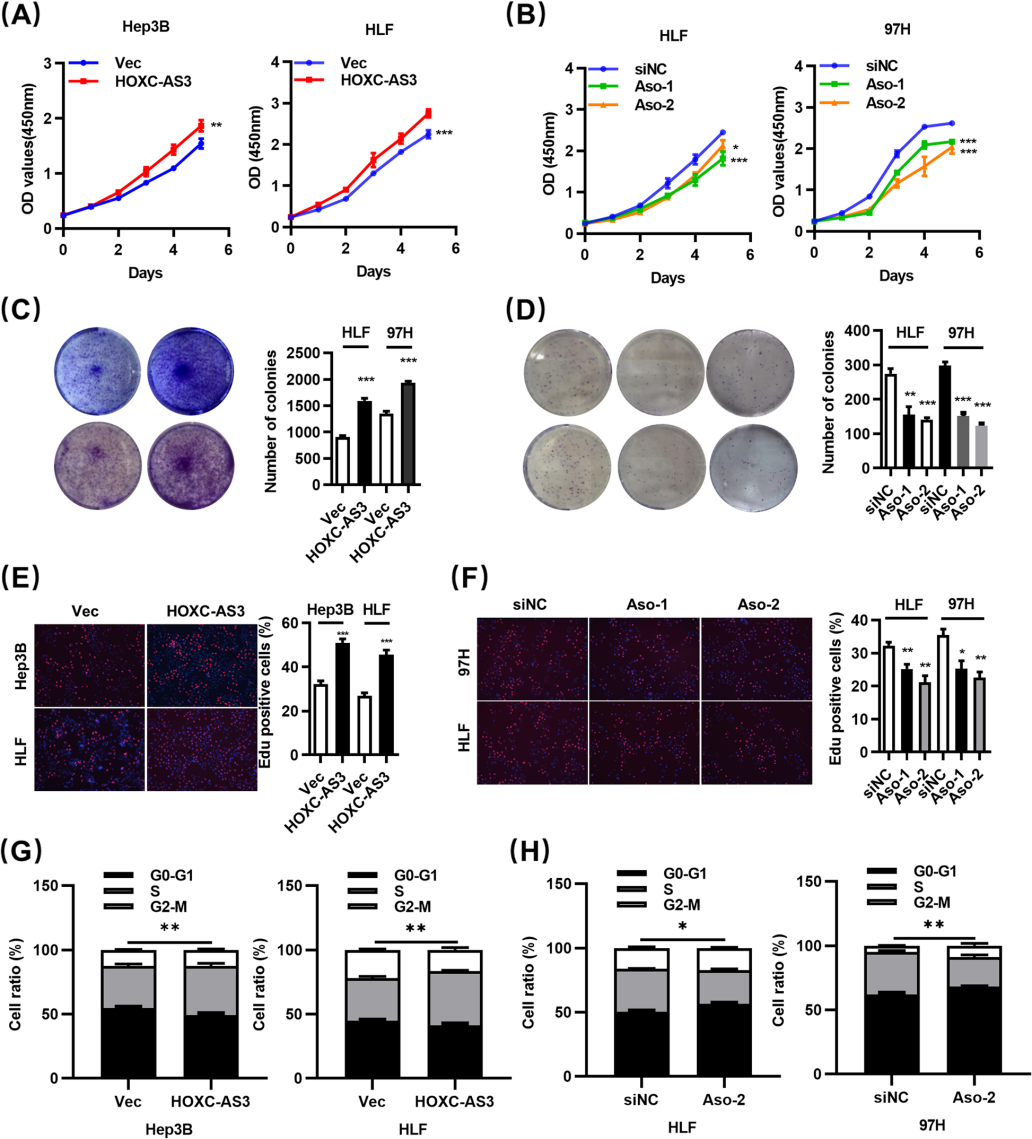
HOXC-AS3 promotes HCC cell proliferation and cell cycle progression in vitro. **A**-**B** CCK-8 assays in HLF, Hep3B and 97H cells with silencing/overexpression of HOXC-AS3. **C**-**D** Colony formation assays in HLF, Hep3B and 97H cells with silencing/overexpression of HOXC-AS3. **E**-**F** EdU assays in HLF, Hep3B and 97H cells with silencing/overexpression of HOXC-AS3. **G**-**H** Cell cycle assays analysis in HLF, Hep3B and 97H cells with silencing/overexpression of HOXC-AS3. Data represent the MEAN ± S.E.M. of three independent experiments. *P < 0.05; **P < 0.01; ***P < 0.001; ****P < 0.0001 (Student’s t test)

To investigate the biological function of HOXC-AS3 in HCC cells, we performed the CCK8 assay and colony formation assays to evaluate the effect of HOXC-AS3 on the growth of HCC cells. Both CCK8 assays and colony formation assay indicated the overexpression of HOXC-AS3 led to efficiently enhanced cell viability in HLF and Hep3B cells. (Fig. 2A, C), whereas knockdown of HOXC-AS3 decreased cell viability in HLF and 97H cells (Fig. 2B, D). We also performed EdU cell proliferation assay, the percentage of S-phase cells was determined by measuring EdU incorporation. As predicted, the population of EdU-positive cells was markedly increased in HLF and Hep3B cells (Fig. 2E). In contrast, knockdown of HOXC-AS3 decreased the population of EdU-positive cells in HLF and 97H cells (Fig. 2F).

As is known, cellular proliferation is tightly associated with cell cycle progression. These results showed that change of HOXC-AS3 expression levels correlated with cell proliferation. Therefore, we hypothesized that HOXC-AS3 might regulate cell cycle progression. Consistently, cell cycle assays further confirmed that HOXC-AS3 promotes cell cycle transition, overexpression of HOXC-AS3 had a decreased rate of G1 phase cells and an increased rate of S-phase cells in HLF and Hep3B cells (Fig. 2G, Fig. S1A), In contrast, knockdown of HOXC-AS3 resulted in an evident cell cycle arrest at the G1/G0 phase in HLF and 97H cells (Fig. 2H, Fig. S1B).

The results above demonstrated that HOXC-AS3 promoted the proliferation and cell cycle progression of HCC cells in vitro.

### HOXC-AS3 promotes HCC cell proliferation in vivo

To further explore whether HOXC-AS3 influences tumor growth in vivo, we established subcutaneous xenograft tumor and orthotopic xenograft tumor models in nude mice using HLF cells which were stably overexpressing HOXC-AS3 and vector,97 cells which stably knockdown HOXC-AS3 and shNC. After 21 days, we found that the subcutaneous tumor volumes of the HOXC-AS3 group were significantly bigger than those of the Vec group (Fig. 4A1, A2), The Aso-2 group were significantly smaller than the shNC group. Statistical analysis of tumor weight and volume on the final day showed that the tumors in the HOXC-AS3 group were much bigger than those in the Vector group, and the Aso-2 group was much smaller than shNC group (Fig. 4B1, B2). In addition, IHC analysis was conducted to determine whether HOXC-AS3 affected the expression of Ki-67 in xenograft tumor tissues. The results showed that the expression of Ki-67 was significantly upregulated in the HOXC-AS3 group, and downregulated in the Aso-2 group (Fig. 4C1, C2). Moreover, we also measured CDK2 expression in xenograft tumors by WB and the results showed that CDK2 was increased in the HOXC-AS3 group, and decreased in the Aso-2 group (Fig. 4D1, D2, E1 and E2). Next, we analyzed the volume of liver orthotopic xenograft tumors and the result showed that the overexpression of HOXC-AS3 greatly promoted tumor growth (Fig. 4F1, G1, H1), knockdown of HOXC-AS3 greatly inhibited tumor growth (Fig. 4F2, G2, H2).

Taken together, these results demonstrated that HOXC-AS3 could promote the proliferation of HCC cells in vivo.

### HOXC-AS3 interacts with CDK2

**Fig. 3:**
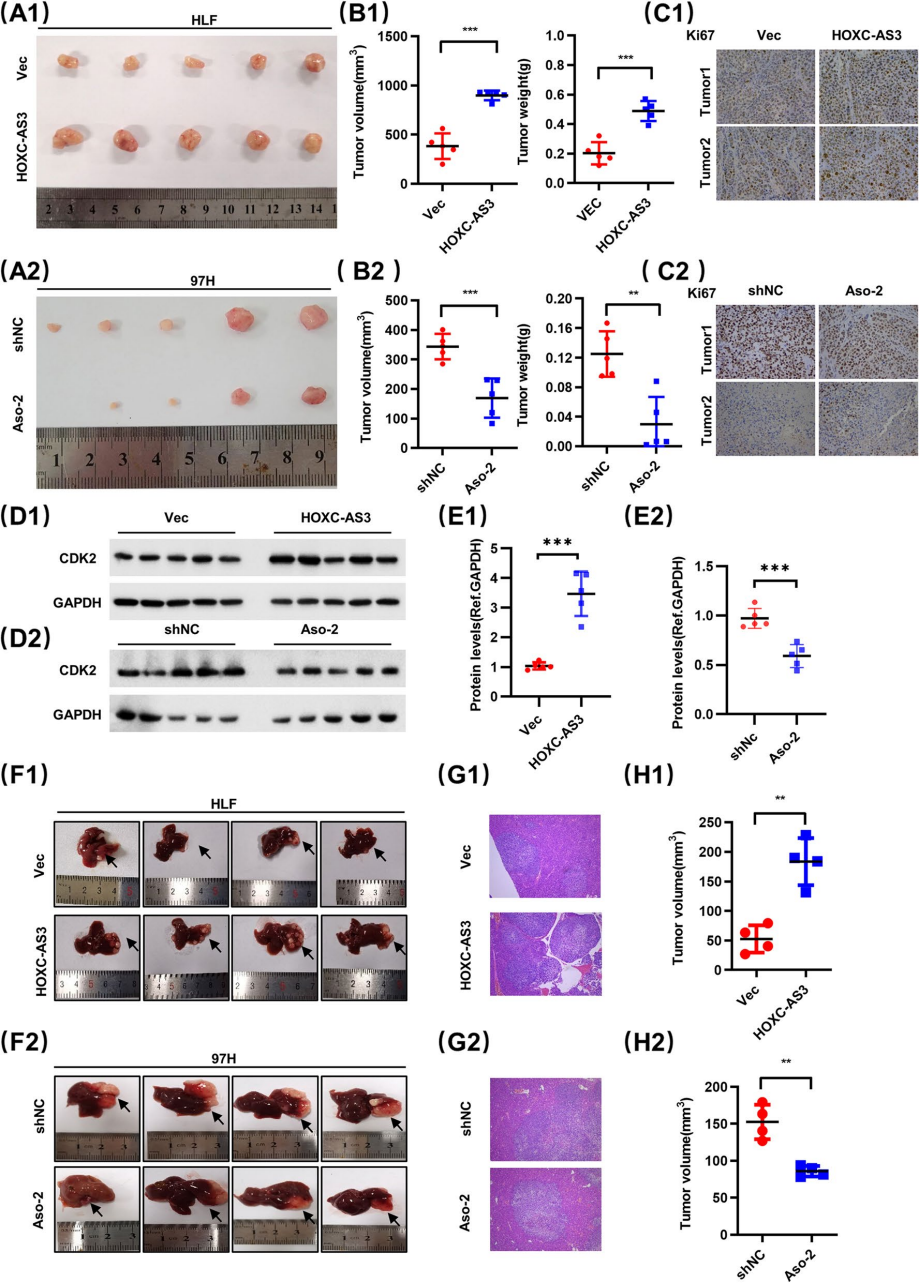
HOXC-AS3 promotes HCC cell proliferation in vivo. (**A1**) Representative images of subcutaneous xenograft tumors formation of the Vec group and HOXC-AS3 group. The dissected tumors from two groups were photographed. (**A2**) Representative images of subcutaneous xenograft tumors formation of the shNC group and Aso-2 group. The dissected tumors from two groups were photographed. (**B1**) Volumes and Weights of subcutaneous xenograft tumors in Vec group and HOXC-AS3 group. (**B2**) Volumes and Weights of subcutaneous xenograft tumors in shNC group and Aso-2 group. (**C1**) IHC staining Ki-67 expression of subcutaneous xenograft tumors in Vec group and HOXC-AS3 group. (**C2**) IHC staining Ki-67 expression of subcutaneous xenograft tumors in shNC group and Aso-2 group. (**D1**) Western blot show CDK2 expression of subcutaneous xenograft tumors in Vec group and HOXC-AS3 group. (**D2**) Western blot show CDK2 expression of subcutaneous xenograft tumors in shNC group and Aso-2 group. (**E1**) CDK2 levels measured by western blot and gray level in Vec group and HOXC-AS3 group. (**E2**) CDK2 levels measured by western blot and gray level in shNC group and Aso-2 group. (**F1**) Representative images of orthotopic xenograft tumors formation of the Vec group and HOXC-AS3 group. The dissected tumors from two groups were photographed. (**F2**) Representative images of orthotopic xenograft tumors formation of the shNC group and Aso-2 group. The dissected tumors from two groups were photographed. (**G1**) Sections of the Vec group and HOXC-AS3 group xenograft tumors stained with hematoxylin and eosin (H & E). (**G2**) Sections of the shNC group and Aso-2 group xenograft tumors stained with hematoxylin and eosin (H & E). (**H1**) Volumes of subcutaneous xenograft tumors in Vec group and HOXC-AS3 group. (**H2**) Volumes of subcutaneous xenograft tumors in shNC group and Aso-2 group. Data represent the MEAN ± S.E.M of three independent experiments. *P < 0.05; **P < 0.01; ***P < 0.001; ****P < 0.0001 (Student’s t test)

To investigate the mechanisms of HOXC-AS3, we first used subcellular fractionation location assays, which revealed a significant increase in HOXC-AS3 expression in the nucleus compared to the cytosol (Fig. 3A), implying that HOXC-AS3 may play a key regulatory role on the transcriptional level by interacting with nucleus molecules or proteins. The HOXC-AS3-associated protein complex was next studied in HLF cells using an RNA pull-down assay followed by proteomic analysis. The RNA-protein complex HOXC-AS3 was isolated, and mass spectrometry was used to determine the protein’s identification (Fig. 3B). CDK2 was identified among the highly enriched proteins by western blotting from three separate RNA pull-down tests. CDK2 drew our attention due to its well-known function in carcinogenesis. And this remarkable protein was a known cyclin-dependent kinase, with other unique peptides discovered in this MS research. Additionally, the qRT-PCR showed that overexpression or knockdown of HOXC-AS3 had no effects on the expression of CDK2 on the transcriptional level, respectively (Fig. 3C, D), but HOXC-AS3 had effects on the protein level of CDK2. These results indicated that HOXC-AS3 regulated the expression of CDK2 on the post-transcriptional level. Furthermore, the localization of HOXC-AS3 and CDK2 in HLF, 97H, and Hep3B cells was verified by FISH. The confocal results indicated that CDK2 was expressed in the nucleus of HLF, 97H, and Hep3B cells, while HOXC-AS3 was also expressed mainly in the nucleus of HLF, 97H, and Hep3B cells, and was mainly positioned in the nucleus (Fig. 3F).

**Fig. 4:**
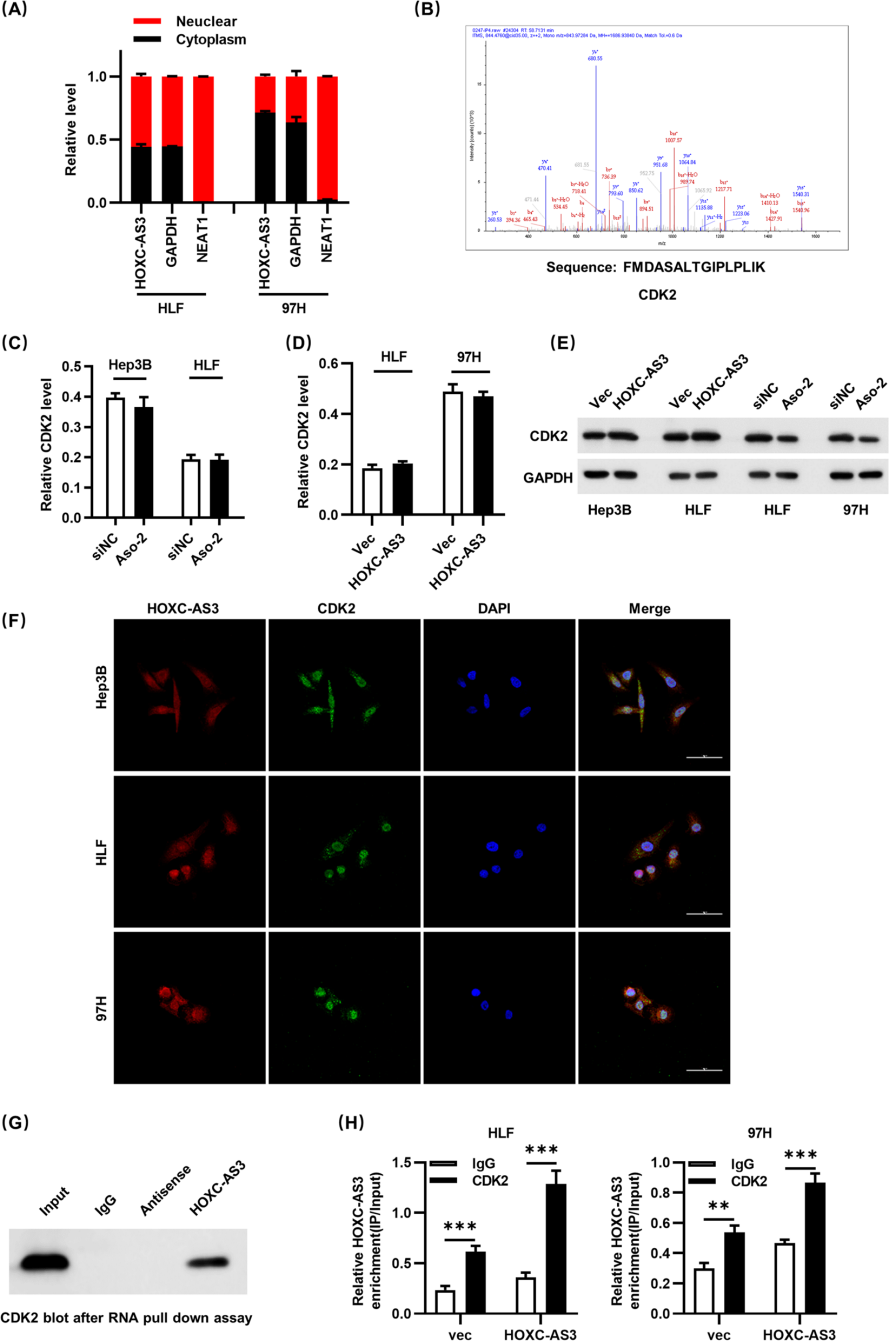
HOXC-AS3 Interacts with CDK2. **A** Relative level of HOXC-AS3 in the nuclear and cytoplasmic fractions of HLF and 97H cells. **B** HOXC-AS3 interacting with CDK2 peptides in the mass spectrum results after RNA pulldown assay. **C** Knockdown of HOXC-AS3 did not significantly inhibit CDK2 expression on transcription level in HLF and Hep3B cells. **D** Overexpressing of HOXC-AS3 did not significantly inhibit CDK2 expression on transcription level in HLF and 97H cells. **E** Protein level of CDK2 in HLF, Hep3B and 97H cells after transfected with Aso-HOXC-AS3 or pcDNA-HOXC-AS3 plasmids. **F** FISH assay to verify the expression localization of HOXC-AS3 and CDK2. **G** RNA pull-down assay to confirm the association between HOXC-AS3 and CDK2. **H** RIP assay to confirm the association between HOXC-AS3 and CDK2 in HLF and 97H cells. Data represent the MEAN ± S.E.M of three independent experiments. *P < 0.05; **P < 0.01; ***P < 0.001; ****P < 0.0001 (Student’s t test)

We used an RNA pull-down technique followed by western blotting using CDK2 antibodies to confirm the physical connection between HOXC-AS3 and CDK2. Our findings revealed that tagged HOXC-AS3 RNA preferentially extracted CDK2 from HLF cell extracts, but not empty vector or antisense HOXC-AS3 (Fig. 3G). Then, RIP assay was conducted with Flag antibody in vector or HOXC-AS3 overexpressing HLF and 97H cells. Subsequently, the enriched RNA was analyzed by qRT–PCR. Results showed that CDK2 could directly bind HOXC-AS3 (Fig. 4H).

### HOXC-AS3 correlates with CDK2-mediated cell cycle progression

**Fig. 5:**
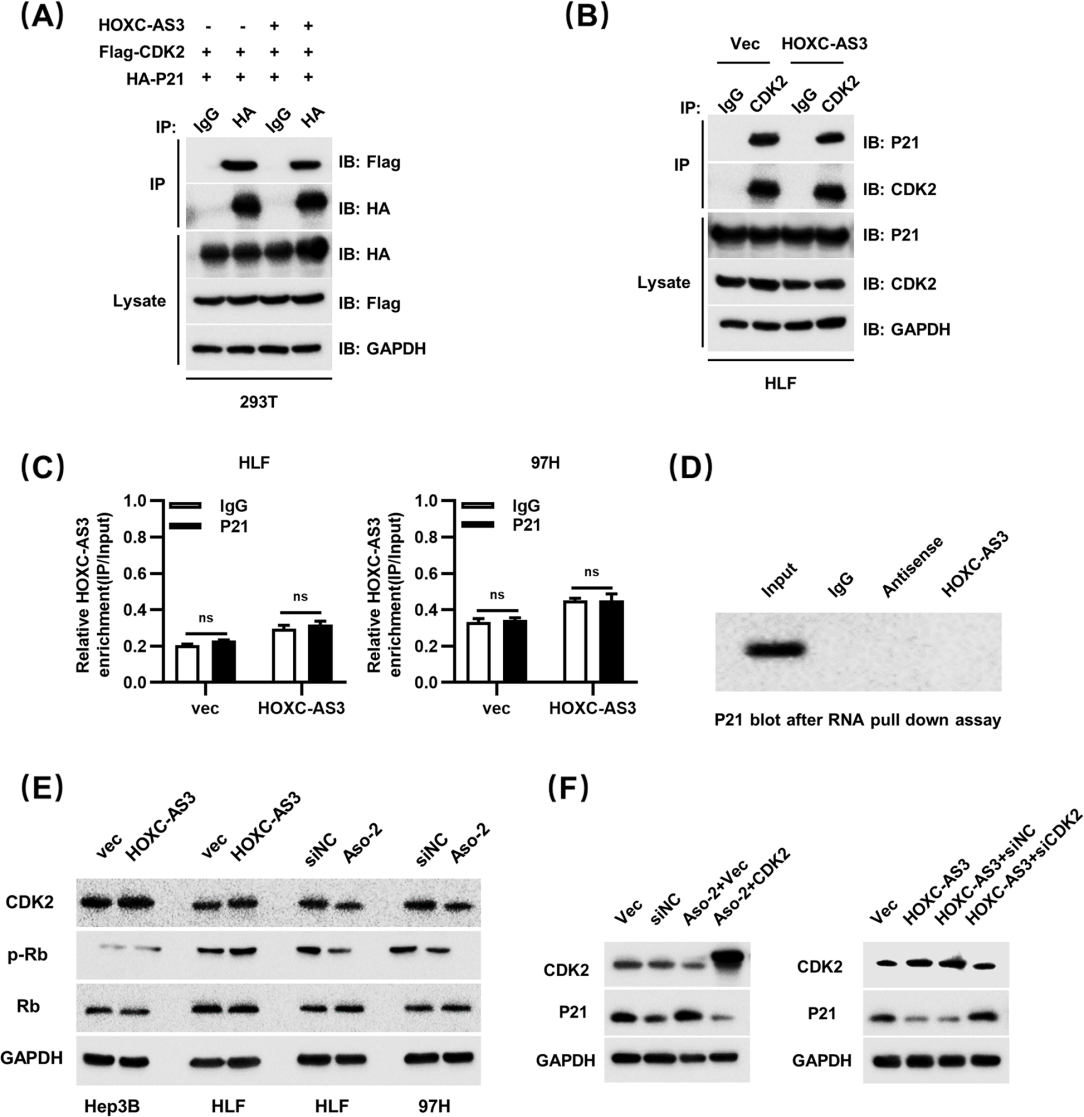
HOXC-AS3 correlates with CDK2-mediated cell cycle progression. **A** Total lysates from HEK293T cells expressing HA-p21 and Flag-CDK2 in the presence of HOXC-AS3 were subjected to IP with HA Ab, followed by western blotting using the indicated Abs. GAPDH, HA-P21 and Flag-CDK2 were used as a loading control. **B** Total lysates from HLF cells in the presence of HOXC-AS3 were subjected to IP with CDK2 Ab, followed by western blotting using the indicated Abs. GAPDH, p21 and CDK2 were used as a loading control. **C** RIP assay to confirm the association between HOXC-AS3 and P21 in HLF and 97H cells. **D** RNA pull-down assay to confirm the association between HOXC-AS3 and P21. **E** Western blot analysis of CDK2, Rb, and phosphorylated Rb in controls, oe-HOXC-AS3, siNC and Aso-HOXC-AS3 groups. **F** Western blot analysis of CDK2 and p21 with siNC, Aso-HOXC-AS3, Aso-HOXC-AS3 + pcDNA-vec and Aso-HOXC-AS3 + pcDNA-CDK2 groups in HLF cells. Western blot analysis of CDK2 and p21 with oe-vec, oe-HOXC-AS3, oe-HOXC-AS3 + siNC and oe-HOXC-AS3 + siCDK2 groups in Hep3B cells. Data represent the MEAN ± S.E.M of three independent experiments. *P < 0.05; **P < 0.01; ***P < 0.001; ****P < 0.0001 (Student’s t test)

The above results demonstrated that HOXC-AS3 regulated the cell cycle. Next, we aimed to study the specific underlying mechanisms. In previous studies, p21 was a critical determinant of G1 arrest by inhibiting CDK2 activation. Hence, we hypothesized that HOXC-AS3 may influence the interaction between p21 and CDK2. To further investigate the correlations among HOXC-AS3, CDK2, and p21, we performed coimmunoprecipitation (co-IP) experiments. In the presence of HOXC-AS3, we found that less Flag-CDK2 interacted with HA-p21(Fig. 5A). Consistently, when HOXC-AS3 was upregulated in HLF cells, less p21 was pulled down by CDK2 (Fig. 5B). Then, we performed RIP assay and RNA Pulldown assay to explore whether HOXC-AS3 could bind to P21. Unfortunately, the results show that p21 could not bind to HOXC-AS3(Fig. 5C, D). Meanwhile, western blot analysis showed that overexpression of HOXC-AS3 could induce the phosphorylation of Rb. Conversely, silencing of HOXC-AS3 could reduce the phosphorylation of Rb (Fig. 5E). These results suggested that HOXC-AS3 promoted the phosphorylation of Rb by interfering with the interaction between CDK2 and p21. To verify the relationship among HOXC-AS3, CDK2, and p21, we performed rescue experiments. Western blot assay exhibited that p21 expression was increased in HLF transfected with Aso-HOXC-AS3. However, the co-transfection of CDK2 and Aso-HOXC-AS3 rescued the repressive role of Aso-HOXC-AS3 in the expression of p21. In contrast, oe-HOXC-AS3 decreased p21 expression, and p21 expression could be rescued by si-CDK2 (Fig. 5F).

Taken together, these results indicate that HOXC-AS3 can speed up the cell cycle by reducing the binding of P21 to CDK2 and increasing the phosphorylation level of RB.

### HOXC-AS3 promotes HCC progression through CDK2

**Fig. 6:**
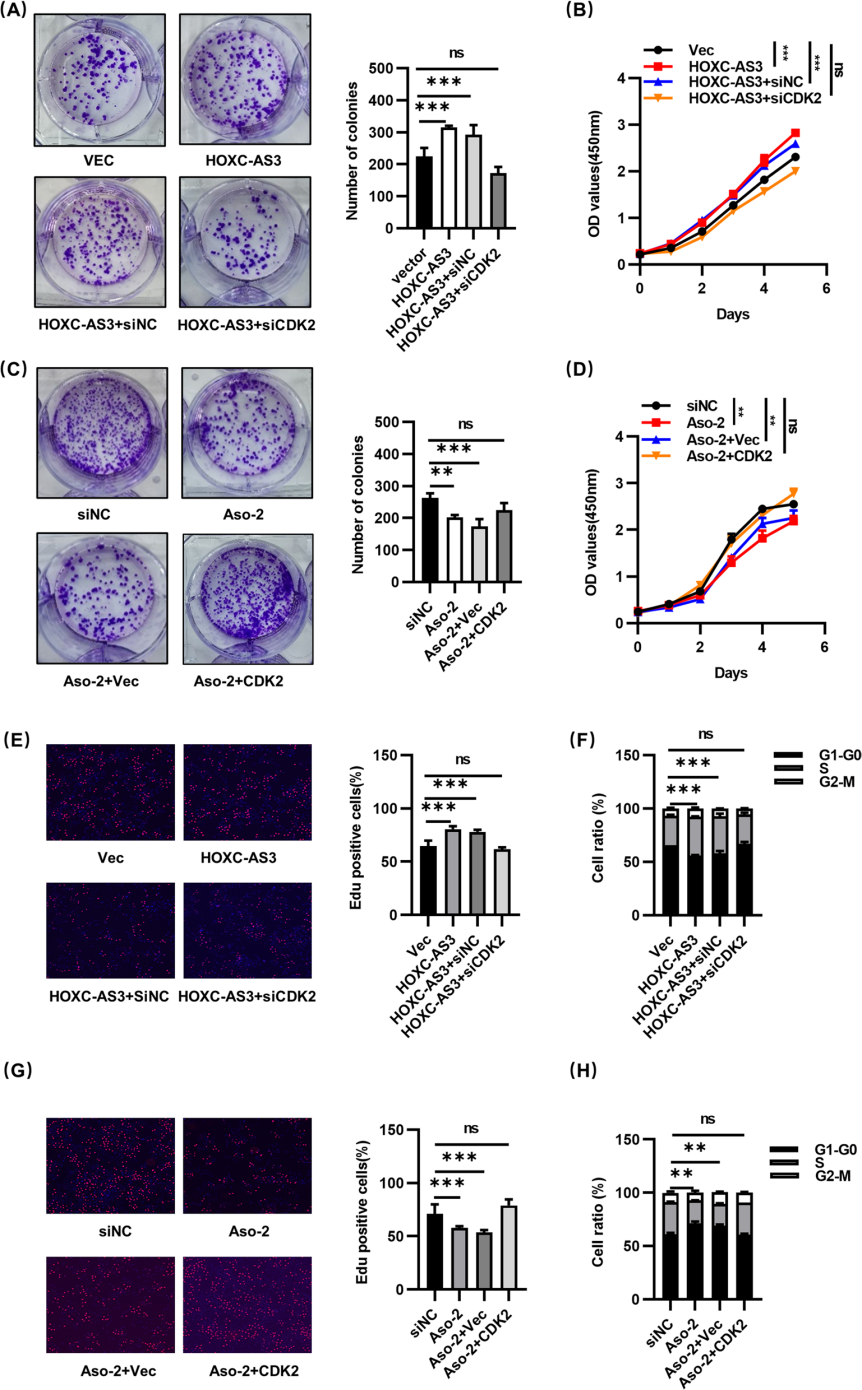
HOXC-AS3 functions through CDK2. **A** Colony formation assays with oe-vec, oe-HOXC-AS3, oe-HOXC-AS3 + siNC and oe-HOXC-AS3 + siCDK2 groups in Hep3B cells. **B** CCK-8 assays with oe-vec, oe-HOXC-AS3, oe-HOXC-AS3 + siNC and oe-HOXC-AS3 + siCDK2 groups in Hep3B cells. **C** EdU assays with oe-vec, oe-HOXC-AS3, oe-HOXC-AS3 + siNC and oe-HOXC-AS3 + siCDK2 groups in Hep3B cells. **D** Cell cycle assays analysis with oe-vec, oe-HOXC-AS3, oe-HOXC-AS3 + siNC and oe-HOXC-AS3 + siCDK2 groups in Hep3B cells. **E** Colony formation assays with siNC, Aso-HOXC-AS3, Aso-HOXC-AS3 + pcDNA-vec and Aso-HOXC-AS3 + pcDNA-CDK2 groups in HLF cells. **F** CCK-8 assays with siNC, Aso-HOXC-AS3, Aso-HOXC-AS3 + pcDNA-vec and Aso-HOXC-AS3 + pcDNA-CDK2 groups in HLF cells. **G** EdU assays with siNC, Aso-HOXC-AS3, Aso-HOXC-AS3 + pcDNA-vec and Aso-HOXC-AS3 + pcDNA-CDK2 groups in HLF cells. **H** Cell cycle assays analysis with siNC, Aso-HOXC-AS3, Aso-HOXC-AS3 + pcDNA-vec and Aso-HOXC-AS3 + pcDNA-CDK2 groups in HLF cells. Data represent the MEAN ± S.E.M of three independent experiments. *P < 0.05; **P < 0.01; ***P < 0.001; ****P < 0.0001 (Student’s t test)

To explore whether CDK2 mediated the regulative effects of HOXC-AS3 on HCC cells, we transfected CDK2 siRNA and siNC into oe-HOXC-AS3 and control cells, or co-transfected pcDNA3.1-CDK2 and Aso-HOXC-AS3 into HCC cell lines. Colony formation assay and CCK8 assays demonstrated that HOXC-AS3 overexpression promoted the proliferation of HCC cells while the silence of CDK2 abrogated the promoting effects of HOXC-AS3 overexpressing (Fig. 6A, B). Likewise HOXC-AS3 knockdown inhibited the proliferation of HCC cells while restoration of CDK2 abrogated the suppressive effects of HOXC-AS3 silencing (Fig. 6C, D). Consistent with the results, the EdU assay and cell cycle assays showed that more cells overexpressing HOXC-AS3 entered the S-phase than the control cells. The increase in the S-phase ratio by HOXC-AS3 overexpression was partly reversed by silencing CDK2 (Fig. 6E, F and Fig. S2A), Similarly, the decrease in the S-phase ratio by HOXC-AS3 silencing was partly reversed by overexpressing CDK2(Fig. 6G, H and Fig. S2B).

Collectively, these data suggested that HOXC-AS3 promoted HCC cell growth by upregulating the levels of CDK2.

## Discussion

The incidence of hepatocellular carcinoma (HCC) has tripled since 1980, making it the top cause of cancer-related deaths [20]. Despite significant breakthroughs in HCC therapy options, patients with HCC have a poor long-term prognosis due to a lack of understanding of the underlying processes of tumor origin and progression [15]. LncRNA dysregulation is linked to the onset and progression of cancers, implying that they could be used as biomarkers for diagnosis and prognosis, as well as therapeutic targets [21–23]. For example, HULC (highly upregulated in liver cancer), a 1.6-kb oncogenic lncRNA, is overexpressed in HCC. In metastatic liver nodules from colon cancer, there are higher amounts of the HULC transcript. HULC is elevated in both tumors and plasma of HCC patients, suggesting that it could be used as a biomarker for the disease [24–26]. Furthermore, HEIH (high expression in HCC), a 1.6-kb SP1-regulated lncRNA found in the 5q34.3 region, is differently expressed in HCC and is closely linked to HCC recurrence. As a result, it could be used to predict the prognosis of HCC patients [27, 28]. HEIH interacts with EZH2 and regulates EZH2 target genes such as p15, p16, p21, and p57, which influence cell cycle regulation. The HEIH gene is knocked out, which inhibits cell proliferation and tumor growth [28].

As a result, finding new therapeutic targets and understanding the underlying molecular mechanism is becoming more essential. The functional relevance and clinical significance of HOXC-AS3 in malignancies are little understood. HOXC-AS3 is an antisense transcript of HOXC10 that is found on chromosome 12q13.13. HOX genes are required for morphogenesis and development [29, 30], and HOX gene expression dysregulation has been observed in a variety of malignancies [31, 32]. Many lncRNAs are found in HOX genes, and they play a key role in carcinogenesis. HOTAIR, which is likewise found on chromosome 12q13.13 and is an oncogenic lncRNA in a variety of cancers, is an antisense transcript of HOXC11 [12]. HOXC-AS3 was elevated in GC (gastric cancer) in a prior study by Erbao Zhang, and when combined with YBX1 could facilitate GC cell proliferation and migration [14].

After that, we discovered that elevated HOXC-AS3 expression was recognized as an independent predictive factor for HCC patients, therefore we investigated HOXC-AS3 in HCC. We discovered that HOXC-AS3 overexpression considerably increased HCC cell proliferation, whereas HOXC-AS3 interference greatly decreased HCC cell proliferation, implying that HOXC-AS3 promoted HCC cell proliferation (Figs. 2, 3, 4, 5 and 6). HOXC-AS3 promoted tumorigenicity and progression in HCC through modulating CDK2 and p21 to trigger the activation of the Rb/E2F1 pathway, according to mechanistic studies. We looked into whether HOXC-AS3 interacted with cell cycle-associated proteins to learn more about how it controlled cell proliferation and progression. (Fig. 3). MS analysis revealed a close association between HOXC-AS3 and CDK2 in the current investigation. The combination of CDK2 and G1 to S-phase transition is triggered by CDK2. When p21 binds to CDK2, this process may be blocked [33]. HOXC-AS3 interacted with CDK2, according to our findings. HOXC-AS3 may also suppress p21 expression. When CDK2 attaches to cyclin E or cyclin A during the G1/S and M phases, it is activated [34, 35]. The hyperphosphorylated Rb boosted the transcription of E2F target genes, E2Fs and other signals then pushed the production and activation of CDK2–CycE and CDK2–CycA complexes, which led to DNA replication and further phosphorylation of Rb, as shown in Fig. 5. As a result, the shift from G1 to S-phase was accelerated [36], CDK2 was considerably downregulated and the commencement of the transition from G1 to S-phase was similarly suppressed after HOXC-AS3 knockdown, which explained the G1 arrest of HCC cells after HOXC-AS3 knockdown.

**Fig. 7:**
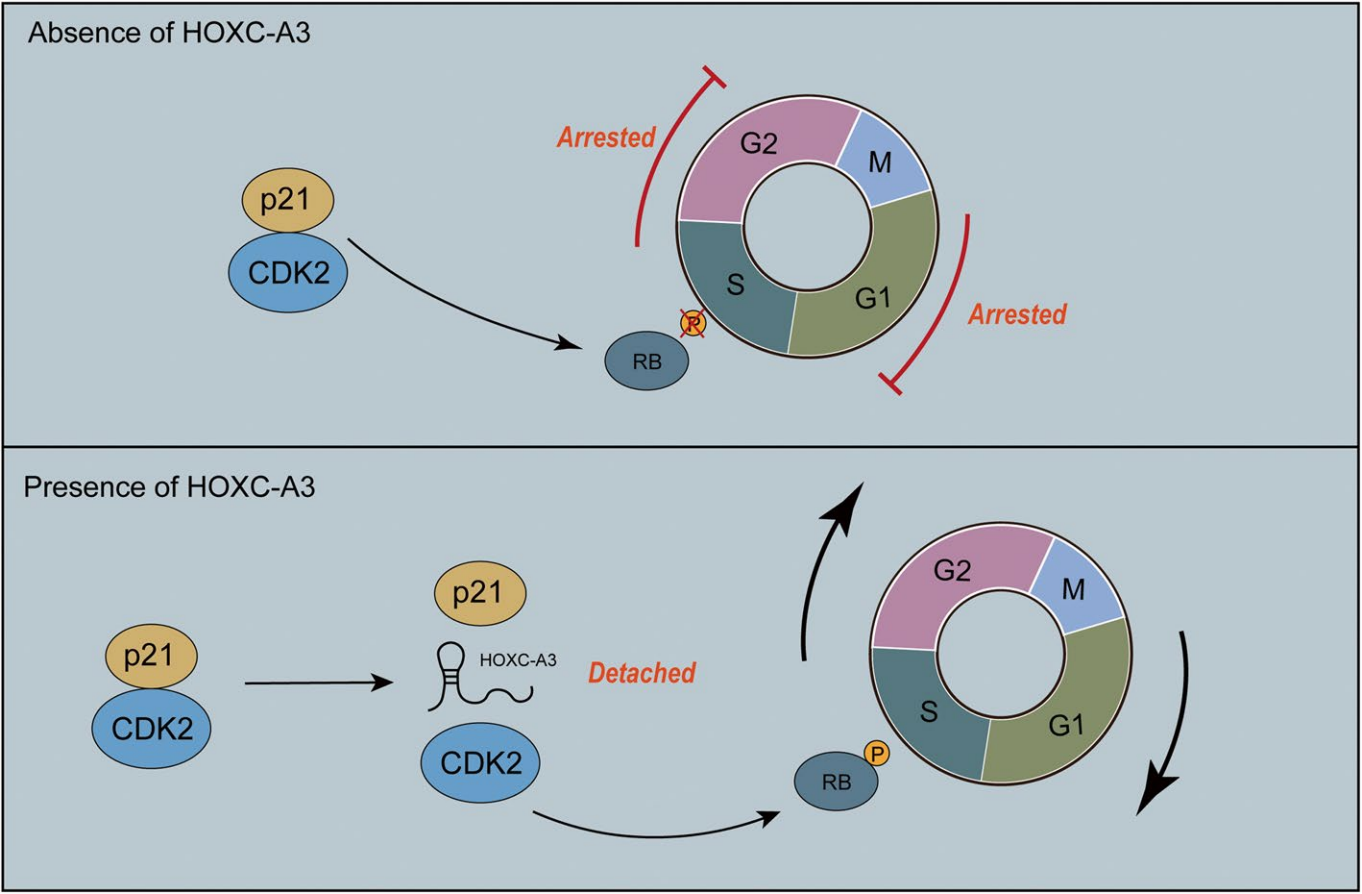
Schematic diagram of the mechanism by which HOXC-AS3 promotes HCC progression. HOXC-AS3 prevents P21 from binding to CDK2, resulting in CDK2 maintaining kinase activity, further phosphorylating RB, and promoting cell cycle from G1 to S-phase, which in turn leads to enhanced cell proliferation, and promoting the progression of HCC

The most important pathological manifestation of cancer is uncontrolled cell division and out-of-control cell cycles. Therefore, it has been expected that our understanding of the principles of cell cycles will guide the effective clinical treatment of cancer [8]. In particular, the CDK family, which regulates the cell cycle, was expected to be a key therapeutic target [37]. When DNA is damaged or exposed to external pressure, the tumor suppressor gene p53 is activated, causing the CDK2 inhibitor p21 to be expressed transiently. It either causes a brief G1 cell cycle stop or a long-term condition of senescence, which is a sort of genome guardianship [38]. Differentiation, apoptosis, transcription, DNA repair, autophagy, and the onset of senescence are all regulated by p21 [39]. As a result, p21 is regarded as one of the most important determinants of cell survival and a potential target for targeted cancer therapy. In this study, we discovered that HOXC-AS3 might bind to CDK2 in HCC, blocking p21-mediated suppression of HCC cell progression. CDK2 expression was drastically reduced when HOXC-AS3 was knocked down. As a result, we hypothesized that HOXC-AS3 binds to CDK2 and inhibits p21-CDK2 interaction. Furthermore, the interaction between HOXC-AS3 and CDK2 had a significant impact on the cell cycle and encouraged the advancement of HCC (Fig. 7).

## Conclusions

Increased HOXC-AS3 expression was found to be an unfavorable sign for HCC patient survival. In HCC cells, HOXC-AS3 controlled proliferation, cell cycle, and tumorigenicity. According to in-depth mechanistic research, HOXC-AS3 binds to CDK2, resulting in increased CDK2 expression and decreased p21 expression, followed by Rb phosphorylation. Overall, the findings of this study revealed new information about the mechanism of carcinogenesis in HCC, as well as a critical biomarker for diagnosis and a possible target for HCC treatment.

## Supplementary Information

**Additional file 1: Figure s1**. (A) Flow Cytometry original picture in HLF, Hep3B cells with Vec and HOXC-AS3 in Cell cycle assays. (B) Flow Cytometry original picture in 97H cells with siNC and Aso-2 in Cell cycle assays.

**Additional file 2: Figure s2**. (A) Flow Cytometry original picture with vec, HOXC-AS3, HOXC-AS3 + siNC and HOXC-AS3 + siCDK2 groups in Hep3B cells in Cell cycle assays. (B) Flow Cytometry original picture with siNC, Aso-2, Aso-2 + pcDNA-vec and Aso-2 + pcDNA-CDK2 groups in HLF cells in Cell cycle assays.

## Data Availability

The datasets used in this study are available upon reasonable request from the corresponding author.

## Abbreviations

HCC: Hepatocellular carcinoma
LncRNA: Long non-coding RNA
qRT-PCR: Quantitative Real-time PCR
Co-IP: Co-inmunoprecipitation
CDK2: Cyclin dependent kinase 2
GC: Gastric carcinoma
siRNA: Small interfering RNA
CCK-8: Cell counting kit-8
EdU: 5-ethynyl-2′ -deoxyuridine
