# Supplementary figures and images for: Long noncoding RNA HOXC-AS3 interacts with CDK2 to promote proliferation in hepatocellular carcinoma
Source: Biomark Res. 2022 Aug 28;10:65. doi: 10.1186/s40364-022-00411-2 (PMC9420287; doi:10.1186/s40364-022-00411-2)

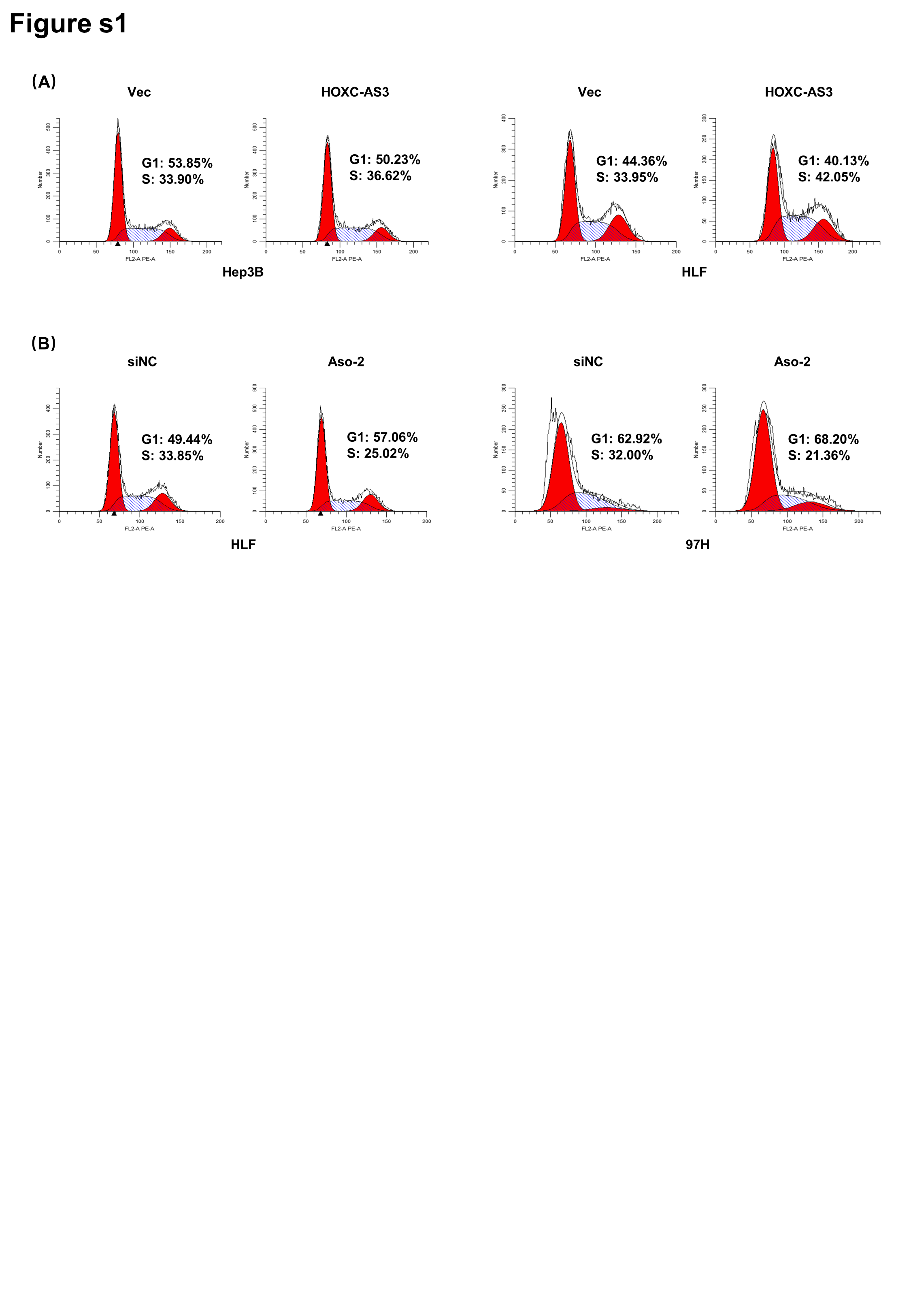

Supplement: Supplementary file 1 — Additional file 1: Figure s1. (A) Flow Cytometry original picture in HLF, Hep3B cells with Vec and HOXC-AS3 in Cell cycle assays. (B) Flow Cytometry original picture in 97H cells with siNC and Aso-2 in Cell cycle assays. [file 40364_2022_411_MOESM1_ESM.tif]

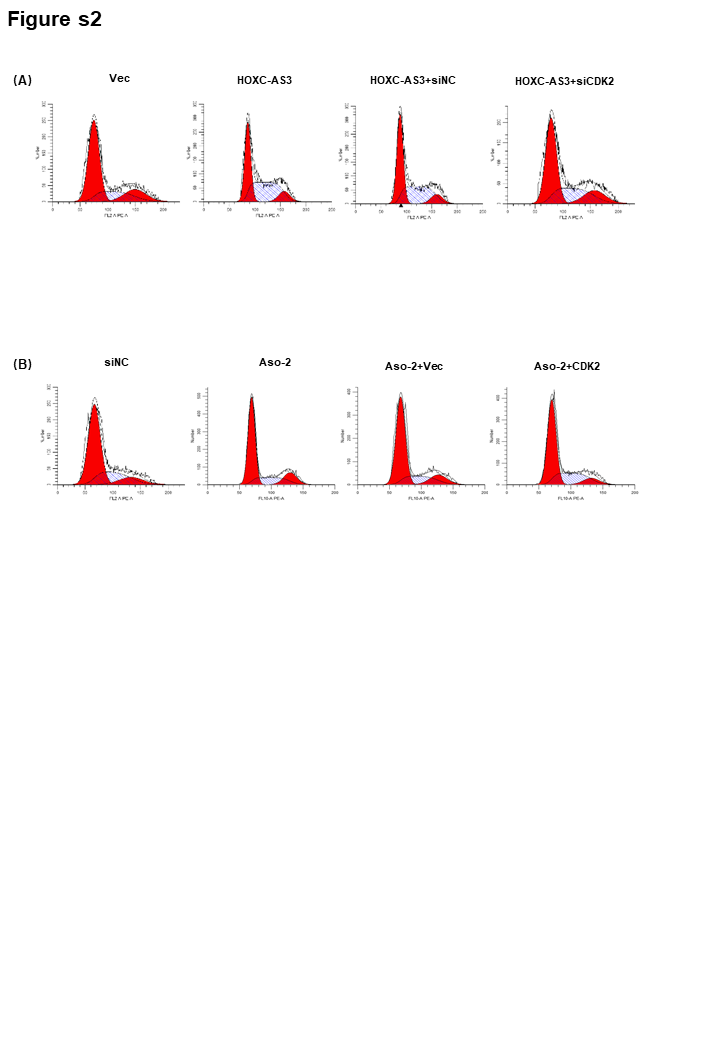

Supplement: Supplementary file 2 — Additional file 2: Figure s2. (A) Flow Cytometry original picture with vec, HOXC-AS3, HOXC-AS3 + siNC and HOXC-AS3 + siCDK2 groups in Hep3B cells in Cell cycle assays. (B) Flow Cytometry original picture with siNC, Aso-2, Aso-2 + pcDNA-vec and Aso-2 + pcDNA-CDK2 groups in HLF cells in Cell cycle assays. [file 40364_2022_411_MOESM2_ESM.tif]
